# Resistance associated mutations to protease inhibitors on HIV-2 infected patients in Portugal

**DOI:** 10.1080/07853890.2021.1896142

**Published:** 2021-09-28

**Authors:** J. Lopes, F. Gonçalves, J. Cabanas, I. Costa, S. Fernandes, I. Diogo, M. Pingarilho, P. Gomes

**Affiliations:** a Centro de Investigação Interdisciplinar Egas Moniz (CiiEM), Egas Moniz Cooperativa de Ensino Superior, Caparica, Portugal; b Laboratório de Biologia Molecular, LMCBM, SPC.HEM – Centro Hospitalar Lisboa Ocidental, Lisboa, Portugal; c Global Health and Tropical Medicine (GHTM), Instituto de Higiene e Medicina Tropical/Universidade Nova de Lisboa (IHMT/UNL), Lisboa, Portugal

## Abstract

**Introduction:**

The second agent of Acquired Immunodeficiency Syndrome was identified as the Human Immunodeficiency Virus type 2 (HIV-2). This virus is endemic to West Africa, but some cases have been reported in European countries with connections to that region [1–3]. The most prevalent mutations for the HIV-2 protease region found in the literature are V47A, I50V, I54M, L90M, I82F, I84V [1]. The aim of the study is to determine the resistance associated mutations to PIs and the most prevalent, in the Portuguese population.

**Materials and methods:**

The sample was selected from the population of patients infected with HIV-2 from the VIH-2 REGA database, from Molecular Biology Laboratory, HEM, CHLO which presented protease sequences available, being a total of 1063 sequences included in this study. Resistance mutations were identified using EU HIV-2 Internet Tool v. 08/2015, resource of HIV-GRADE e.V. Algorithm. The data collected was analysed utilising the software IBM SPSS Estatistics 25. None of the participants signed an informed consent due to the anonymity already in place in this database.

**Results:**

In the sample of the study, the infection of HIV-2 represents only individuals infected with virus from Group A. Of the sequences analysed 546 (51.4%) are from female individuals and 515 (48.4%) from male individuals. The most prevalent mutations were L90M that was present in 211 sequences (19.8%), I54M in 133 sequences (12.5%), I50V in 82 sequences (7.7%), V47A in 77 sequences (7.2%), I82F in 74 sequences (7.0%) and I84V in 68 sequences (6.4%). According to the data collected, only 20 individuals were drug naïve to protease inhibitors for which no protease mutations were identified.

**Discussion and conclusions:**

The guidelines for HIV-2 antiretroviral therapy in Portugal include only the use of three PIs, Darunavir (DRV), Lopinavir (LPV) and Saquinavir (SQV). With this study we were able to determine that mutations associated with high level resistance to these PIs are related with high impact on saquinavir and lopinavir, maybe because they are available for a longer time than darunavir. L90M was present in almost 20% of the samples and V47A in 7% of the sequences. Nevertheless, I50V with high impact in darunavir was also present in 7.7% of the sequences. Due to very limited options of PIs available for the HIV-2 infection, it is of extreme importance to know the mutations present in this region before choosing a PI for an antiretroviral regimen. Therefore, whenever possible (detectable viral load) it is extremely important to monitor resistance to PIs before starting or switching therapy for HIV-2 infected individuals, in order to achieve maximum efficacy and avoid mutations accumulation.
